# Primary prevention of preeclampsia: myth or reality?

**Published:** 2015-12-30

**Authors:** Julian Alberto Herrera

**Affiliations:** Professor Emeritus of Medicine. Facultad de Salud. Universidad del Valle, Cali, Colombia

Preeclampsia is a disease of the human species, described more than a century ago, of unknown cause, which shares with atherosclerosis risk factors, biochemical abnormalities, endothelial dysfunction and a multifactorial origin. Reducing atherosclerosis lethality is achieved with secondary prevention; and similarly, in preeclampsia, early diagnosis and timely obstetric resolution (secondary prevention) prevents that the mother reaches the end of its natural history, where acute overlay of atherosclerotic plaques in the arteries spiral induces multiple organ failure and increases the maternal risk of death 1. The hospital care scheme implemented in Colombia achieved a reduction in maternal mortality by 9%, and when complemented with a preventive prenatal outpatient scheme (Biopsychosocial Model) designed to prevent progression of endothelial dysfunction, the reduction of maternal mortality increased up to 25% 2.

For decades, different approaches to the prevention of preeclampsia were investigated. One proposal was the prescription of acetylsalicylic acid in low doses (75-100 mg / day) to reduce inflammation and improve the balance between thromboxane A2/prostacyclin. With this approach, a reduction of 10% was observed in the probability of preeclampsia appearing in the general obstetric population (OR= 0.90, 95% CI= 0.84 to 0.97), and a 25% reduction in the high-risk obstetric population (OR= 0.75; 95% CI= 0.66 to 0.85) 3. Calcium supplementation was proposed to block the vasoconstrictor effect of parathyroid hormone (PTH). With this strategy, it was observed a decrease of 37% in the probability of having preeclampsia (OR= 0.63, 95% CI= 0.44 to 0.90); but two subsequent studies in which the supplementation began in the second quarter, using the same dose, did not show any protective effect (OR= 0.94, 95% CI= 0.76-1.16). But when the dose (<1g/day elemental calcium) was reduced, it was estimated through nine studios that the probability of preeclampsia decreased up to 62% (OR= 0.38, 95% CI 0.28 to 0.52) 4. 

Most studies evaluated the baseline calcium intake, but not the levels of maternal and placental hormones that regulate its metabolism, nor the circulating levels of ionized calcium. In a pregnant woman who has a high calcium intake and high supplementation, her supplemented calcium will be lost with the body fluids, since these hormones reduce its absorption and/or increase its excretion. In addition to lessening the effect of PTH, a proper balance in the vasomotor tone can be achieved by inducing calcium-dependent enzymes such as phospholipase A_2_ and nitric oxide synthase, responsible for producing two potent vasodilators, nitric oxide and prostaglandin E_2_.

In these studies the bioavailability of calcium was not controlled as a confounding factor. A proposal derived from it was investigated by adding conjugated linoleic acid (CLA) (450 mg/day) to the calcium supplementation (600 mg / day elemental calcium) in low doses in order to reduce lipid concentrations and inflammation 5. This strategy reduced the risk by 80% (OR= 0.20 95% CI= 0.44 to 0.82), taking into account studies in both food intake and the bioavailability of calcium 6.

In the present issue of Colombia Medica, Alzate *et al*. 7, compared calcium citrate supplementation in low doses and conjugated linoleic acid in pregnant women from the second quarter, compared with the use of calcium carbonate alone. They describe a preventive effect for preeclampsia in adolescents (OR= 0.000, 95% CI= 0.00 to 0.44) for the first treatment, compared with the usual scheme (calcium carbonate) (OR= 0.96; 95% CI= 0.73-1.27).

Why did the calcium supplementation with low doses plus linoleic acid show a greater risk reduction than calcium alone at high doses? Several studies in different populations have shown protective mechanisms for risk with the association of low doses of calcium plus linoleic acid by improving the balance between thromboxane A_2_/prostaglandin E_2_, by reducing intracellular free calcium, by antagonizing the activity of the renin-angiotensin-aldosterone, by reducing the endothelial dysfunction, and by reversing the metabolic syndrome; 5,6 which has not been observed with calcium supplementation alone 8.

Why was the risk reduction greater in adolescents than in older age groups? One could speculate that given their young age, they would be less exposed to chronic infections and gestational diabetes, factors associated with oxidative stress and endothelial dysfunction 9; and because of their accelerated growth, the nutritional imbalance could be an important avenue for endothelial dysfunction 10.

Primary prevention of disease is an important strategy because it reduces maternal mortality and prevents the leading cause of intrauterine growth restriction, *i.e.* preeclampsia, which is associated with fetal programming for cardiovascular diseases in the adult 11. There's no doubt that these preventive results are more relevant for African descendants and indigenous ethnic groups, in both of which teenage pregnancy is common, and that have lower attendance to prenatal care and institutional delivery, which would prevent perinatal hypoxia and cognitive deficits of children and adolescents 12. Preventing teen pregnancy and preventing maternal deaths are development goals which break this poverty circle 9. The experience of our country with the Biopsychosocial model 2, has shown that it is possible to achieve the primary prevention of preeclampsia, by reducing the incidence of the disease in western Colombia in a pilot study, and then maternal mortality in the country in a sustainable way 2. The study by Alzate *et al*. 7 makes an important light on the primary prevention of preeclampsia in adolescents. We need that the new generations of Colombians are not anymore exposed to fetal programming for life-threatening diseases in adverse perinatal environments, which very often increases disability and violence 13. This is a challenge that must be undertaken for greater social equity, and to achieve the long-awaited consolidation of peace for Colombians.
